# PhaSePro: the database of proteins driving liquid–liquid phase separation

**DOI:** 10.1093/nar/gkz848

**Published:** 2019-10-15

**Authors:** Bálint Mészáros, Gábor Erdős, Beáta Szabó, Éva Schád, Ágnes Tantos, Rawan Abukhairan, Tamás Horváth, Nikoletta Murvai, Orsolya P Kovács, Márton Kovács, Silvio C E Tosatto, Péter Tompa, Zsuzsanna Dosztányi, Rita Pancsa

**Affiliations:** 1 MTA-ELTE Momentum Bioinformatics Research Group, Department of Biochemistry, Eötvös Loránd University, Budapest H-1117, Hungary; 2 Institute of Enzymology, Research Centre for Natural Sciences of the Hungarian Academy of Sciences, Budapest H-1117, Hungary; 3 Department of Biomedical Sciences, University of Padova CNR Institute of Neuroscience, Padova, Italy; 4 Structural Biology (CSB), Brussels, Belgium; Structural Biology Brussels (SBB), Vrije Universiteit Brussel (VUB), Brussels 1050, Belgium

## Abstract

Membraneless organelles (MOs) are dynamic liquid condensates that host a variety of specific cellular processes, such as ribosome biogenesis or RNA degradation. MOs form through liquid–liquid phase separation (LLPS), a process that relies on multivalent weak interactions of the constituent proteins and other macromolecules. Since the first discoveries of certain proteins being able to drive LLPS, it emerged as a general mechanism for the effective organization of cellular space that is exploited in all kingdoms of life. While numerous experimental studies report novel cases, the computational identification of LLPS drivers is lagging behind, and many open questions remain about the sequence determinants, composition, regulation and biological relevance of the resulting condensates. Our limited ability to overcome these issues is largely due to the lack of a dedicated LLPS database. Therefore, here we introduce PhaSePro (https://phasepro.elte.hu), an openly accessible, comprehensive, manually curated database of experimentally validated LLPS driver proteins/protein regions. It not only provides a wealth of information on such systems, but improves the standardization of data by introducing novel LLPS-specific controlled vocabularies. PhaSePro can be accessed through an appealing, user-friendly interface and thus has definite potential to become the central resource in this dynamically developing field.

## INTRODUCTION

One of the most exciting recent developments in the field of molecular cell biology is the discovery that certain proteins can undergo liquid–liquid phase separation (LLPS) inside the cell, driving the formation of diverse membraneless organelles/biological condensates, such as stress granules, P-bodies, the nucleolus and postsynaptic densities (1–3). These dynamic, non-stoichiometric supramolecular assemblies represent a unique functional and structural level of cellular organization. Their functions often cannot be derived from the functions of individual proteins, but emerge from the collective behaviour of their constituent macromolecules (4). They confer a wide range of functional advantages on cells (5) due to their unique material properties (5,6), and rapid responses to environmental triggers (8). It was also proposed that multiple non-specific weak interactions can more readily emerge and be maintained through evolution than specific strong interactions, and thus liquid condensates exhibit a favourable cost-benefit ratio (7). Ever since the discovery and first analysis of liquid droplets in Drosophila embryos (9), an increasing number of cellular functions have been ascribed to such liquid condensates, including the regulation of most stages of the life cycle of RNAs (10–13), transcriptional regulation and silencing (14,15) and the signal transduction networks of membrane receptors (16,17). LLPS has emerged as a general mechanism of cellular organization, exploited not only by eukaryotic cells, but also by bacteria and viruses (18,19). Besides diverse physiological roles of these dense liquid condensates (1–3), their fundamental roles are also highlighted by the fact that mutations affecting their regulation are often implicated in devastating neurological disorders, such as amyotrophic lateral sclerosis (ALS) and frontotemporal dementia (FTD), and are also associated with cancers and muscular atrophies (20).

The ability to drive liquid–liquid phase separation (LLPS) is encoded in protein sequences, but it can be achieved by diverse functional modules, including disordered regions of low sequence complexity, multivalent domain – motif interactions, RNA binding domains, oligomerization domains and various combinations of these modules (21). As a unifying feature, LLPS is typically driven by multivalent weak interactions, which ensure the dynamic, liquid-like properties of the resulting condensates. Liquid condensates show a broad range of morphologies, shapes, sizes and compositions (2). Some of them are constituted of a single dense phase, while others show intricate core-shell structures, in which multiple immiscible phases are embedded into each other as dictated by their surface tension and viscosity properties (13,22,23). Many have a rounded shape as true liquid droplets (24,25), or form more irregular structures along the surface of membranes (16,17,26). While some only contain a few types of macromolecules (27,28), others, such as P-bodies and stress granules, host hundreds of proteins and thousands of RNA molecules (29–32). It has been proposed that regardless of the size and compositional richness of the condensates, usually only one or a few proteins, referred to as ‘scaffolds’, drive their formation. The other constituents, the so-called ‘client’ proteins, do not substantially contribute to the formation of the condensates. Through interactions with the scaffold, clients are readily compartmentalized by the condensate and often directly promote its characteristic functions (33,34).

While in the last years an avalanche of high-impact publications reported on novel cases of proteins involved in LLPS with the numbers still fast increasing, many open questions remain about the composition of the various condensates, their exact biological roles and modes of regulation, and the sequence characteristics of protein regions that drive LLPS. To a large extent, our limited insight comes from the lack of a comprehensive, curated database of experimentally validated LLPS driver proteins/protein regions. To fill this gap, here we introduce the PhaSePro database that is a comprehensive, carefully curated resource of proteins that have been experimentally demonstrated to drive LLPS.

## INFORMATION AVAILABLE IN PhaSePro

PhaSePro (https://phasepro.elte.hu) is a novel database that provides a wealth of information on LLPS in a structured way. It is a queryable, public database currently containing 121 entries (as of September 2019) collected through comprehensive literature curation. Entries in the database describe proteins/protein regions that were demonstrated to drive LLPS together with the supporting literature references. Proteins can drive phase separation on their own or as part of well-defined multicomponent systems, the two scenarios being clearly distinguished in PhaSePro. PhaSePro currently includes 109 eukaryotic, 5 bacterial and 7 viral entries, well illustrating that the formation of liquid condensates through LLPS is a universal mechanism for creating dynamic subcompartments in the cell that has been demonstrated across different domains of life, as well as viruses.

**Figure 1.**
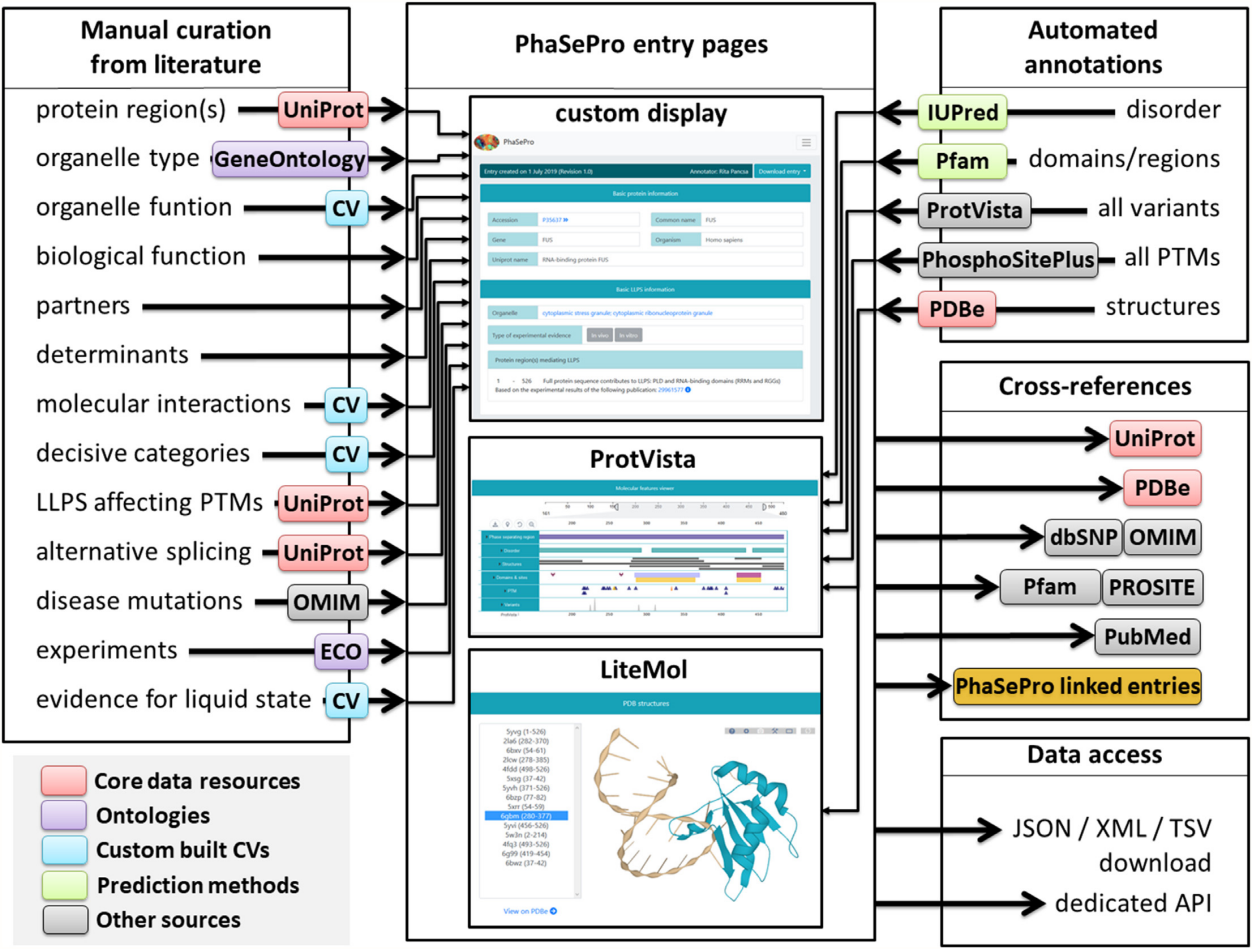
Data integrated into PhaSePro. Manual curation (left) is based on the literature. The biological function of the organelle, the partners required for and other determinants of LLPS are expressed as free text descriptions, while all other annotations are encoded using references to UniProt sequences or as terms in various ontologies and controlled vocabularies (CVs) (see Supplementary Tables). Automated annotations (top right) are added from the outputs of various sequence-based prediction methods (IUPred (38) for disorder prediction and Pfam (39) for conserved protein regions), from ProtVista (40) (for sequence variants omitting predicted variants), from PhosphoSitePlus (37) (for phosphorylation, methylation, acetylation and ubiquitination sites) and from PDBe (41) (for structures overlapping the LLPS driver region). Entries are cross-linked to various data resources and the data contained in PhaSePro can be accessed via download or the dedicated API.

We have collected proteins experimentally verified to drive phase separation *in vivo* and/or *in vitro* from the literature; cases inferred from computational prediction or homology were not included. The core data represented in PhaSePro were derived from manual curation, which are then extended with data automatically retrieved from diverse resources (Figure 1).

The bulk of information from manual annotation is encoded in a structured way through the use of references to existing databases, ontologies and custom-built controlled vocabularies (CVs). The protein sequences investigated in the experiments addressing LLPS are defined using canonical UniProt sequences, while for three entries specific isoforms needed to be used. The sequence boundaries and characteristics of the experimentally validated LLPS driver region(s) are also provided for each entry protein. The exact sequence regions driving LLPS were confirmed for most of the entry proteins, while in some cases only the full-length protein has been subjected to experiments. These scenarios are clearly distinguished in PhaSePro. For the proteins that are parts of multi-component systems being able to drive LLPS only together, PhaSePro defines the accessory proteins needed for LLPS. For all LLPS proteins/systems PhaSePro provides information on the membraneless organelle(s) (MO) formed, expressed via GeneOntology cellular component terms.

Position specific information known or assumed to directly affect LLPS based on the processed literature is specified using the UniProt sequence as reference. These information include post-translational modifications, disease mutations (defined through dbSNP (35) if possible and connected to OMIM (36)), and alternative splicing events. In addition, all other isoforms that contain any sequence changes within the LLPS driver region were also added from UniProt. Functional and molecular aspects of the organelle formation derived from the literature are encoded via purpose-built LLPS-specific CVs (see Supplementary Tables S1–S4 and next section for details).

The biological function of the formed organelle, the molecular determinants and the required molecular partners of LLPS are provided in the form of free-text descriptions. To decide whether a given case of LLPS was partner and/or modification dependent, only experimental parameters close to physiological conditions and protein concentration were considered. Experimental procedures used for the investigation of LLPS are condensed from the relevant papers by the annotators, and are detailed as free text enriched with ontology links (see next section).

Automated annotations also enrich the information provided by PhaSePro. All post-translational modifications (PTMs) that have been described for the full protein including phosphorylation, methylation, acetylation and ubiquitination were taken from PhosphoSitePlus (37); protein disorder predictions from the IUPred2A server (38), and conserved protein region assignations from Pfam (39). Sequence variants are imported through ProtVista (40), with predicted deleterious variants filtered. In addition, all structures that overlap with the LLPS driving region are imported from PDBe (41).

For each protein we have a cross-reference to UniProt entries to include core information, including accessions, gene/protein names and source species. Other featured information are linked to the respective database entries (PDBe, Pfam/PROSITE, OMIM/dbSNP). All information taken from the literature through manual curation is linked to corresponding manuscripts via EuropePMC (42).

## INTRODUCTION OF LLPS-SPECIFIC CONTROLLED VOCABULARIES AND EXTENSIONS TO AVAILABLE ONTOLOGIES

The majority of information in PhaSePro is expressed using controlled vocabularies (CVs) and ontologies to aid the findability of the data and the interoperability of the database, advancing adherence to FAIR principles (43). Using CVs instead of free text for data representation helps the interpretation of data, aids computational analyses, and reduces redundancy of information in databases. Correspondingly, several fields of biology established their respective CVs and ontologies, including biomolecular interactions (44) of post-translational modifications (45). However, CVs have not been developed for the rapidly expanding field of LLPS, and in parallel with the development of PhaSePro, we also laid down the foundations of data standardization by the development of 4 distinct CVs that describe the following four aspects of LLPS and the membraneless organelles formed.

(i) The functional roles of membraneless organelles (MOs)/granules in the cell are defined using eight classes already defined in several dedicated reviews (3–6) (Supplementary Table S1). These include terms such as ‘protective storage/reservoir’ for MOs that store molecules in an inactive state, or ‘activation/nucleation/signal amplification/bioreactor’ for MOs that bring together components of a reaction. (ii) We also introduced a CV of 19 terms for the different molecular interaction types that could contribute to LLPS based on (21), including terms such as ‘multivalent domain-motif interactions’ or ‘coiled-coil formation’ (Supplementary Table S2). (iii) A dedicated CV with 6 terms was defined to describe the molecular determinants and mechanisms that are pertinent to LLPS, such as the ability of the proteins to form membrane clusters or if PTMs are required for the LLPS (Supplementary Table S3). (iv) Finally, the diverse experimental observations that could support the liquid state of the condensates, such as temperature-dependence or the observed dynamic exchange of molecules within the droplet, were grouped in a separate CV of seven terms that was partly built based on the review by Mitrea D *et al.* (46) (Supplementary Table S4). The terms of these CVs are explained in more detail on the About/Help page of PhaSePro.

To ensure the best integration with frequently used ontologies, we reviewed the existing classification of membraneless organelles available in Gene Ontology (GO) (47) (see Supplementary Table S5). Several cellular component GO terms, falling under the ‘non-membrane bounded organelle’ parent term already describe membraneless organelles. However, to make PhaSePro annotations more precise, we created new terms when it was necessary. Experimental procedures used for studying liquid–liquid phase separation were similarly reviewed, connecting them to terms of the Evidence and Conclusion Ontology (48) (ECO—see Supplementary Table S6). The use of GO and ECO are fully in line with the practices of core data resources, such as UniProt, and will enable future integration and standardization efforts.

## IMPLEMENTATION AND SERVER FEATURES

PhaSePro is presented through a DJANGO (version 2.1.1) based web interface, fueled by a multi-layer SQL database, which allows a vast amount of parallel queries to be completed in a fraction of a second. The SQL database contains all the information collected from the literature alongside with protein information derived from UniProt. Each record represents a single protein and is linked to a unique UniProt accession. To maintain the best possible compatibility through the various devices and browsing options users have, the front-end of PhaSePro is represented solely as a combination of bootstrap (version 4.3.1) and JQuery (version 2.1.4).

In addition to access the data through the online interface, data can also be accessed via downloading the data in JSON, XML or TSV formats, or by the RESTful API serving standard JSON format (e.g. https://phasepro.elte.hu/rest/P35637.json). New data currently missing from PhaSePro can be submitted through a dedicated interface found on the ‘Annotate’ page.

## THE INTERFACE

The online interface of PhaSePro offers convenient approaches for users to find relevant data. The web-server opens with a Home page containing general information on LLPS and specific information of PhaSePro, with links to two example entry pages, and a search bar, which can be found on the top of the Browse/Search page as well. The search bars use a completion based method, offering the best matches for the queries. The browse function encompasses a table containing all entries, where users are able to filter the database by various options alongside with the ability to search by keywords or regular expressions. Clicking on any row in either the search bar results or inside the Browse table directs the user to the relevant entry page. Any set of entries compiled through the use of filters can be further customized and downloaded in any of the three available formats (JSON, XML and TSV).

A comprehensive online documentation about the usage and functionalities of PhaSePro are available on the ‘About/Help’ page.

PhaSePro also provides information on candidate proteins that are likely to drive LLPS but their status cannot be fully ascertained by the available experimental data. These entries are collected on the ‘Candidates’ page (https://phasepro.elte.hu/candidates), which is presented in a similar fashion as the ‘Browse’ page, with the same functionalities, including the option of downloading them. The ‘Statistics’ page provides various statistics about the database, including the fraction of entries with *in vivo*/*in vitro* support, taxonomic distributions of proteins, or the frequencies of various terms in the four developed CVs.

## ENTRY PAGES

**Figure 2.**
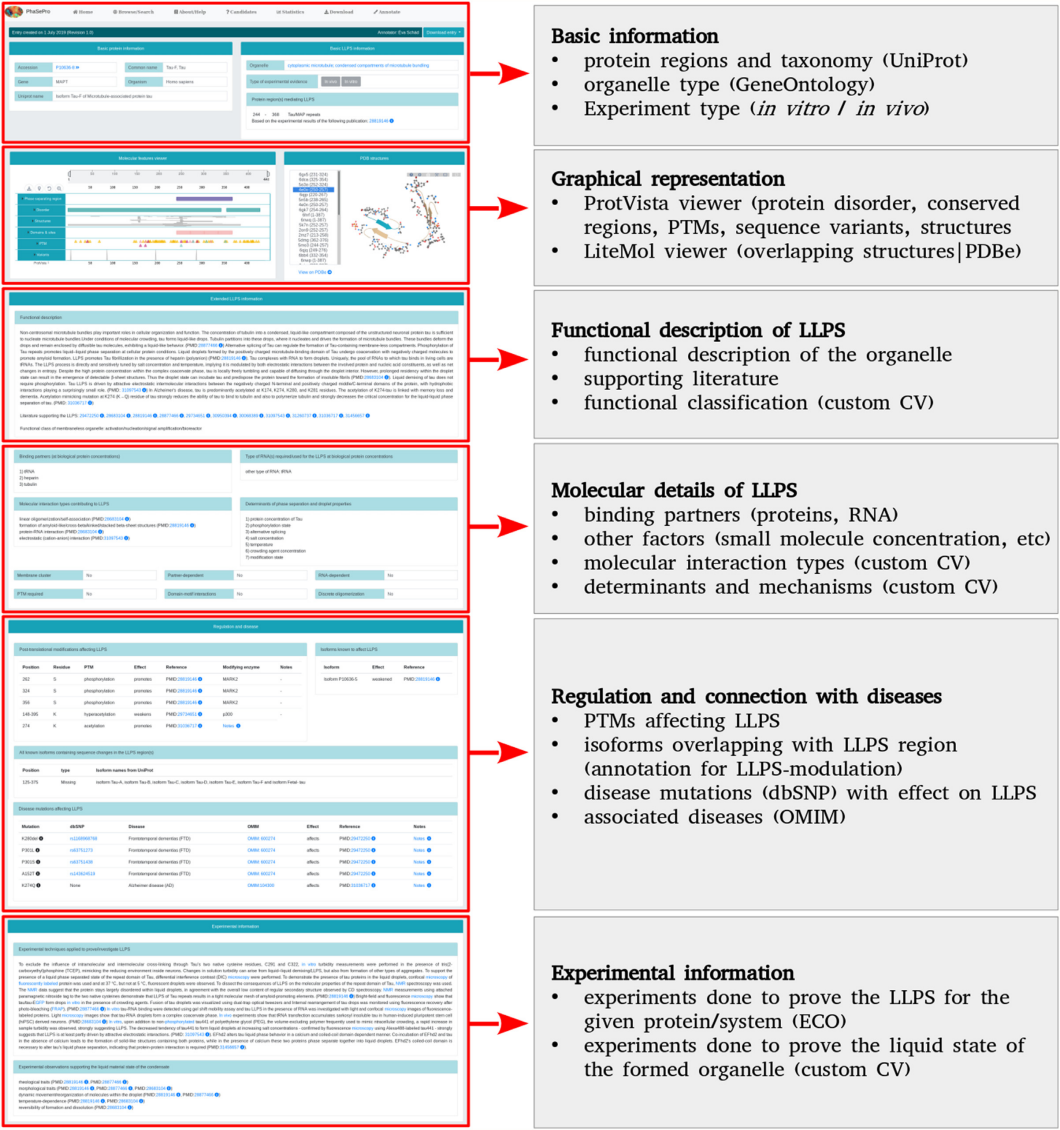
Entry pages in PhaSePro. Each entry page is structured to display information pertaining to specific aspects of the liquid–liquid phase separation (LLPS) or the formed membraneless organelles. The figure uses the entry of human Tau protein (https://phasepro.elte.hu/entry/P10636–8).

Each entry in PhaSePro correspond to a single protein, and has a dedicated entry page detailing all relevant information collected either manually from the literature or in an automated way from source databases (Figure 2).

The first section of the entry page contains the basic information on the protein undergoing LLPS and the formed organelle. The top bar contains information on the release of the given entry and the annotator(s), and provides the option of downloading the annotation of the given entry in JSON, TSV and XML formats. Also, it is indicated here when the given entry is not a self-sufficient LLPS driver, but is part of a multi-component system in which the collective behaviour of several proteins are required for LLPS to occur. This bar is followed by core information on the given entry protein from UniProt (49) (UniProt accession, common name, gene name, Ensembl transcript ID, species name, NCBI taxon ID, and protein name). On the right side, the PhaSePro core information is provided, namely the specific membraneless organelle connected to the GO cellular components system, the specification of the type of experimental evidence (*in vitro*, *in vivo* or both), the joined entries (if the given entry is part of a multicomponent LLPS system) and specific sequence region(s) that drive LLPS, including UniProt sequence boundaries and a description of the domain/compositional/disorder properties of the given sequence region supported by literature reference.

These core sections are followed by a section for the graphical representation of sequence- and structure-level information connected to the protein. For sequence-level annotations PhaSePro applies the recently released smart visualization tool of UniProt, ProtVista (40) that enables mapping of UniProt-annotated features onto the entry proteins, including domain and site information, variations and disease-associated mutations, and many more. To fit with the requirements specific for our database, the molecular features viewer was extended with our annotated LLPS regions. As many regions driving LLPS are intrinsically disordered and are regulated by posttranslational modifications (PTMs), disorder prediction by IUPred (38), domain predictions by PfamScan (50) and PTMs from PhosphoSitePlus (37) were also incorporated into the graphical representation. We provide an overview of the PDB structures overlapping with the corresponding LLPS protein regions, if available, together with a LiteMol structure viewer (51) for their visualization. The viewer provides an interactive cartoon-style structural view for the selected overlapping PDB structure in beige color with the region(s) overlapping the annotated LLPS protein region(s) highlighted in turquoise in one of the chains belonging to the entry protein.

The visualization tools are followed by a larger block of ‘Extended LLPS information’ incorporating two separate sections. The first section gives a description of the functional relevance and distinctive features of the given LLPS system using a free-text description distilled by the annotators based on the relevant literature. Together with the description, a list of related articles is also provided. To represent the functional class(es) of the formed MO in a more structured way, it is also defined using the custom built CV (Supplementary Table S1).

The second part of the extended LLPS section provides information on the partners and other determinants known to be required for, or to promote or negatively regulate LLPS. As several known LLPS events are known to depend on the presence of RNA, a dedicated block specifies what type of RNA(s) (if any) are involved. If known, the molecular interaction types playing a role in driving the given LLPS process are also listed using the CV described in Supplementary Table S2. At the end of this section, categorical classifications of the given LLPS system are provided using the CV described in Supplementary Table S3.

The ‘Regulation and disease’ section provides detailed information on the PTMs and alternative splicing events demonstrated to affect LLPS by experiments. Both types of data are organized into a strict format, defining the sequence change together with other supporting information defining the effect the change has on LLPS and cross-references to supporting literature. Apart from isoforms directly affecting LLPS, this section also provides alternative splicing-derived isoforms that have not yet been shown to have a direct effect on LLPS, but do contain sequence changes within the annotated LLPS region, and thus may show an altered LLPS behaviour compared to the canonical isoform. Disease mutations, whose effects on LLPS have been experimentally investigated (if any) are also listed using the sequence variant nomenclature (52), crosslinked to dbSNP, together with the related diseases crosslinked to OMIM.

Furthermore, each entry page contains a block of ‘Experimental information’ that contains an extensive textual description of the LLPS-specific experiments performed that is enriched with links to associated ECO ontology terms. Finally, a list of evidence that support the liquid state of the given condensate backed by literature references is given using the CV detailed in Supplementary Table S4.

## CONCLUSIONS

PhaSePro is a database of proteins driving liquid–liquid phase separation that aims to provide an up-to-date view on the variety of biological condensates that rely on LLPS, including their major architectural properties, functions and regulation. It is carefully curated and incorporates experimentally validated LLPS drivers from all kingdoms of life. We are convinced that PhaSePro will greatly benefit the scientific community by providing (i) a freely accessible, easy-to-use, organized resource with all relevant data on LLPS proteins, (ii) the basis for standardization of experimental approaches and functional characterization, (iii) crucial data for furthering the elucidation of the sequence determinants and molecular mechanisms enabling liquid–liquid phase separation and (iv) a high-quality training set for the development of new methods targeting the computational identification of novel LLPS proteins.

By storing the most comprehensive list of phase separation driver proteins published so far, supplied with detailed annotation on the biological relevance and regulation of condensates, PhaSePro has a definite potential to become the central resource in this fast-expanding field. To achieve this goal we are dedicated to ensure the long-term availability of the database.

## DATA AVAILABILITY

Our aim is to maintain a regularly updated online resource with periodic releases at least twice a year, by incorporating previously published LLPS literature. To successfully accomplish this goal, we also kindly encourage the scientific community to submit newly identified phase separation driver proteins to PhaSePro using our detailed downloadable annotation guidelines and sample document or the more simple online submission form (https://phasepro.elte.hu/annotate). We also encourage authors to contact us if they have published new information on the already existing entries. As PhaSePro is located in the EU, data collection from users and submitters through the server is executed via a secure interface using HTTPS, and fully adheres to the General Data Protection Regulation (GDPR).

## Supplementary Material

gkz848_Supplemental_Files
